# Genome Sequence of *Lysobacter* sp. Strain BMK333-48F3, the Producer Strain of Potent Lipopeptide Antibiotics of the Tripropeptin Family

**DOI:** 10.1128/MRA.00969-21

**Published:** 2021-12-09

**Authors:** Patricia Arlt, Hideki Hashizume, Masayuki Igarashi, Harald Gross

**Affiliations:** a Department of Pharmaceutical Biology, Pharmaceutical Institute, University of Tübingen, Tübingen, Germany; b Department of Microbiology, Institute of Microbial Chemistry, Tokyo, Japan; c German Centre for Infection Research, Partner Site Tübingen, Tübingen, Germany; University of Maryland School of Medicine

## Abstract

*Lysobacter* sp. strain BMK333-48F3 is known primarily for its production of the antibiotically active tripropeptins. Here, we report its draft genome sequence, which will give insight into the biosynthesis of tripropeptins and enable genome mining for further secondary metabolites.

## ANNOUNCEMENT

*Lysobacter* sp. strain BMK333-48F3 was isolated from a soil sample collected at Naha, Okinawa Prefecture, Japan (1), and was deposited at the National Institute of Advanced Industrial Science and Technology (accession number FERM BP-7477). It is known for the production of tripropeptin antibiotics (1–6). The members of this lipopeptide class represent potent antibiotics that are highly active against drug-resistant Gram-positive organisms by interfering with cell wall biosynthesis (7, 8). In order to investigate the complete biosynthetic capacity for secondary metabolism, to locate the biosynthetic locus of the tripropeptins, and to clarify the taxonomic position of the strain, the genome of BMK333-48F3 was sequenced at BaseClear (Leiden, The Netherlands).

BMK333-48F3 was harvested from a 1-day culture grown at 27°C and 180 rpm in LB (9), and its genomic DNA (gDNA) was isolated using a gDNA extraction minikit (RBC Bioscience, UK). The gDNA was sheared using a Covaris g-TUBE device; a 10-kb Pacific Biosciences (PacBio) genomic library was generated according to the manufacturer’s recommendations, including size selection using the BluePippin size selection system (Sage Science, Inc.), and sequenced on a PacBio RS II instrument using one single-molecule real-time (SMRT) cell. An aliquot of the gDNA obtained was used to create a genomic Nextera XT paired-end library for sequencing using an Illumina HiSeq 2500 platform. The results from both sequencing platforms enabled a *de novo* hybrid assembly. The initial quality assessment of the Illumina data was based on data passing the Illumina chastity filter. Chastity is defined as the brightest base intensity divided by the sum of the brightest and second brightest base intensities. Clusters of reads pass the filter if no more than 1 base call has a chastity value below 0.6 in the first 25 cycles. This filtration process removes the least reliable clusters from the image analysis results (also see reference 10).

Afterward, fastq-mcf, from the ea-utils v1.1.2 package (https://bio.tools/ea-utils), was used for trimming of the Illumina adapters. For phiX removal, Bowtie v2.6.1 (11) was applied to align the reads against the genome of coliphage phiX174 (GenBank accession number NC_001422.1). The unaligned reads were used for downstream analyses. Further quality assessment was based on the remaining reads using FastQC v0.11.5 (12), and low-quality bases were trimmed using BBDuk in BBMap v36.77 (13). The resultant high-quality reads were assembled into contigs using ABySS v2.0.2 (14). The PacBio data were processed and filtered using the SMRT Link software suite, whereby subreads shorter than 50 bp were discarded. Subsequently, the long reads were mapped to the draft assembly using BLASR v1.3.1 (15). These alignments enabled the linkage of contigs, which were in turn placed into scaffolds. The orientation and order of and distance between the contigs were estimated using SSPACE-LongRead v1.0 (16), and gapped regions were closed using GapFiller v1.10 (17). Finally, assembly errors and the nucleotide disagreements between the Illumina reads and the scaffold sequences were corrected using Pilon v1.21.1 (18). All software settings were kept at their defaults. Overall, the hybrid genome assembled *de novo* consists of three scaffolds and comprises 5.2 Mb. Annotation was conducted with the Prokaryotic Genome Annotation Pipeline (PGAP) v5.2 pipeline (19, 20), while an automated secondary metabolism analysis was performed using antiSMASH v6.0.1 (21). Essential genome features are summarized in Table 1.

**TABLE 1:** Sequencing metrics for *Lysobacter* sp. strain BMK333-48F3

Parameter	Finding for *Lysobacter* sp. strain BMK333-48F3
PacBio sequencing	
No. of reads	1,452,682
Mean read length (bp)	3,450
No. of mapped reads	1,061,956
Avg coverage (×)	790
Illumina sequencing	
Read length (nucleotides)	2 × 150
No. of reads	7,259,870
Yield (Mbp)	870
Avg quality score	37.95
Avg coverage (×)	162
Median insert size (bp)	320
*De novo* hybrid assembly	
Genome size (bp)	5,227,231
GC content (%)	69.7
No. of contigs	7
No. of scaffolds	3
* N*_50_ (bp)	5,224,492
No. of gaps	4
Avg gap size (bp)	352
Total no. of genes	4,487
No. of coding genes	4,395
No. of predicted biosynthetic gene clusters	12

### Data availability.

The genome sequence has been deposited in the NCBI GenBank database under accession number JAIHOO000000000. The corresponding raw sequencing data sets have been deposited in the NCBI SRA database under accession numbers SRX11855925 (Illumina reads) and SRX11855924 (PacBio reads).
